# Transcriptional Heterogeneity of *Cryptococcus gattii* VGII Compared with Non-VGII Lineages Underpins Key Pathogenicity Pathways

**DOI:** 10.1128/mSphere.00445-18

**Published:** 2018-10-24

**Authors:** Rhys A. Farrer, Christopher B. Ford, Johanna Rhodes, Toni Delorey, Robin C. May, Matthew C. Fisher, Elaine Cloutman-Green, Francois Balloux, Christina A. Cuomo

**Affiliations:** a MRC Centre for Global Infectious Disease Analysis, Department of Infectious Disease Epidemiology, School of Public Health, Imperial College London, London, United Kingdom; b Infectious Disease and Microbiome Program, The Broad Institute of MIT and Harvard, Cambridge, Massachusetts, USA; c UCL Genetics Institute, University College London, London, United Kingdom; d Institute of Microbiology and Infection & School of Biosciences, University of Birmingham, Birmingham, United Kingdom; e Department of Microbiology, Virology & Infection Prevention & Control, Great Ormond Street Hospital NHS Foundation Trust, London, United Kingdom; Carnegie Mellon University

**Keywords:** *Cryptococcus*, capsule, ergosterol, host response, host-pathogen interactions, laccase, mRNA

## Abstract

Cryptococcus gattii is a pathogenic yeast of humans and other animals which causes disease predominantly in immunocompetent hosts. Infection begins when aerosolized yeast or spores enter the body, triggering an immune response, including engulfment by macrophages. To understand the early transcriptional signals in both the yeast and its mammalian host, we performed a time-course dual-transcriptome sequencing (RNA-seq) experiment for four lineages of C. gattii (lineages VGI to IV) interacting with mouse macrophages at 1, 3, and 6 h postinfection. Comparisons of *in vitro* to *ex vivo* gene expression levels indicated that lineage VGII is transcriptionally divergent from non-VGII lineages, including differential expression of genes involved in capsule synthesis, capsule attachment, and ergosterol production. Several paralogous genes demonstrated subfunctionalization between lineages, including upregulation of capsule biosynthesis-related gene *CAP2* and downregulation of *CAP1* in VGIII. Isolates also compensate for lineage-specific gene losses by overexpression of genetically similar paralogs, including overexpression of capsule gene *CAS3* in VGIV, which have lost the *CAS31* gene. Differential expression of one in five C. gattii genes was detected following coincubation with mouse macrophages; all isolates showed high induction of oxidative-reduction functions and downregulation of capsule attachment genes. We also found that VGII switches expression of two laccase paralogs (from *LAC1* to *LAC2*) during coincubation of macrophages. Finally, we found that mouse macrophages respond to all four lineages of C. gattii by upregulating FosB/Jun/Egr1 regulatory proteins at early time points. This report highlights the evolutionary breadth of expression profiles among the lineages of C. gattii and the diversity of transcriptional responses at this host-pathogen interface.

**IMPORTANCE** The transcriptional profiles of related pathogens and their responses to host-induced stresses underpin their pathogenicity. Expression differences between related pathogens during host interaction can indicate when and how these genes contribute to virulence, ultimately informing new and improved treatment strategies for those diseases. In this paper, we compare the transcriptional profiles of five isolates representing four lineages of C. gattii in rich media. Our analyses identified key processes, including those involving cell capsule, ergosterol production, and melanin, that are differentially expressed between lineages, and we found that VGII has the most distinct profile in terms of numbers of differentially expressed genes. All lineages have also undergone subfunctionalization for several paralogs, including capsule biosynthesis and attachment genes. Most genes appeared downregulated during coincubation with macrophages, with the largest decrease observed for capsule attachment genes, which appeared to be coordinated with a stress response, as all lineages also upregulated oxidative stress response genes. Furthermore, VGII upregulated many genes that are linked to ergosterol biosynthesis and switched from expression of the laccase *LAC1* to expression of *LAC2 ex vivo*. Finally, we saw a pronounced increase in the FosB/Jun/Egr1 regulatory proteins at early time points in bone marrow-derived macrophages, marking a role in the host response to C. gattii. This work highlights the dynamic roles of key C. gattii virulence genes in response to macrophages.

## Abstract (precis)

The transcriptional profiles of related pathogens and their responses to host-induced stresses underpin their pathogenicity. Expression differences between related pathogens during host interaction can indicate when and how these genes contribute to virulence, ultimately informing new and improved treatment strategies for those diseases. In this paper, we compare the transcriptional profiles of five isolates representing four lineages of C. gattii in rich media. Our analyses identified key processes, including those involving cell capsule, ergosterol production, and melanin, that are differentially expressed between lineages, and we found that VGII has the most distinct profile in terms of numbers of differentially expressed genes. All lineages have also undergone subfunctionalization for several paralogs, including capsule biosynthesis and attachment genes. Most genes appeared downregulated during coincubation with macrophages, with the largest decrease observed for capsule attachment genes, which appeared to be coordinated with a stress response, as all lineages also upregulated oxidative stress response genes. Furthermore, VGII upregulated many genes that are linked to ergosterol biosynthesis and switched from expression of the laccase *LAC1* to expression of *LAC2 ex vivo*. Finally, we saw a pronounced increase in the FosB/Jun/Egr1 regulatory proteins at early time points in bone marrow-derived macrophages, marking a role in the host response to C. gattii. This work highlights the dynamic roles of key C. gattii virulence genes in response to macrophages.

## INTRODUCTION

Infectious diseases impose a huge burden on human society. In recent years, fungi have gained widespread attention for their ability to threaten both animal and plant species on a global scale (1, 2). However, many features of fungal genomes and transcriptomes that enable the infection of diverse hosts and ecological niches remain largely unexplored, especially for emerging pathogens (3, 4). One such example is the basidiomycete yeast Cryptococcus gattii, which can cause pneumonia and meningoencephalitis in humans (5). While C. gattii causes less overall global morbidity than its sibling species C. neoformans, lineages have emerged with hypervirulent clinical phenotypes, a process that was most strikingly observed in the Pacific Northwest of the United States in the late 1990s (6). C. gattii is comprised of four genetically distinct lineages designated the VGI to VGIV molecular types, among which lineage VGII has been found to be associated with the highly virulent subtypes seen in the United States. These lineages are sufficiently divergent to include 737 lineage-specific gains and/or losses of genes across all four lineages (approximately 4% of the genes in any given isolate), including DNA transposons and genes involved in the response to oxidative stress and import into mitochondrial inner membrane (7).

In addition to genetic differences between lineages, changes in gene regulation underlie important morphological and physiological traits in fungal pathogens (8, 9). However, the mechanisms of gene regulation, evolution of gene networks, and rewiring of transcriptional modules within lineages remain largely uncharacterized for many infectious diseases, including those caused by C. gattii. New virulent lineages can emerge through mutation and/or recombination events (10), which in turn lead to different transcriptional profiles and enhanced virulence profiles, such as that of the hypervirulent C. gattii VGIIa sublineage in the Pacific Northwest (11), which descends from two alpha mating-type parents (6) and is characterized by enhanced intracellular parasitism (11).

Previous studies have demonstrated unique expression profiles among different isolates of *Cryptococcus* under various conditions. For example, in C. neoformans reference isolate H99, genes encoding membrane transporters for nutrients, general metabolism, and oxidative stress response have been shown to be upregulated in the presence of macrophages (12) and amoebae (13). Janbon et al. also characterized differential expression of transporters, transcription factors, and genes involved in lipid metabolism in C. neoformans in comparisons between rich media, limited/starved media, and pigeon guano (14). In addition to analyzing protein-coding gene expression, Janbon et al. also identified nearly 1,200 miscellaneous RNAs that may perform a range of functions that include morphogenesis via small open reading frames (ORFs) or noncoding RNA (ncRNA) with structural or regulatory roles. Concurrently, Chen et al. described an increase in genes involved in metabolic processes, alkaline response, salt tolerance, and oxidative stress for two C. neoformans isolates grown in cerebral spinal fluid compared to growth in rich media (15).

Few studies have focused on gene expression in C. gattii, although, notably, Ngamskulrungroj et al. identified an upregulation of laccase genes involved in melanin formation, and of other genes involved in cell wall assembly and metabolism in C. gattii VGIIa outbreak strain R265, relative to the nonoutbreak C. gattii VGIIb strain in minimal medium (16). In this report, we extend the description of C. gattii gene expression levels to characterize variation across all four major lineages of C. gattii, including the outbreak strain VGIIa R265 and a nonoutbreak isolate, ENV152, also from the VGIIa subgroup, both *in vitro* and at three early time points (1, 3, and 6 h) following coincubation and engulfment by murine bone marrow-derived macrophages (BMDMs). Furthermore, we describe concurrent expression changes in mouse macrophages during C. gattii coincubation, demonstrating the potential for the simultaneous profiling of both host and pathogen responses.

## RESULTS

**FIG 1:**
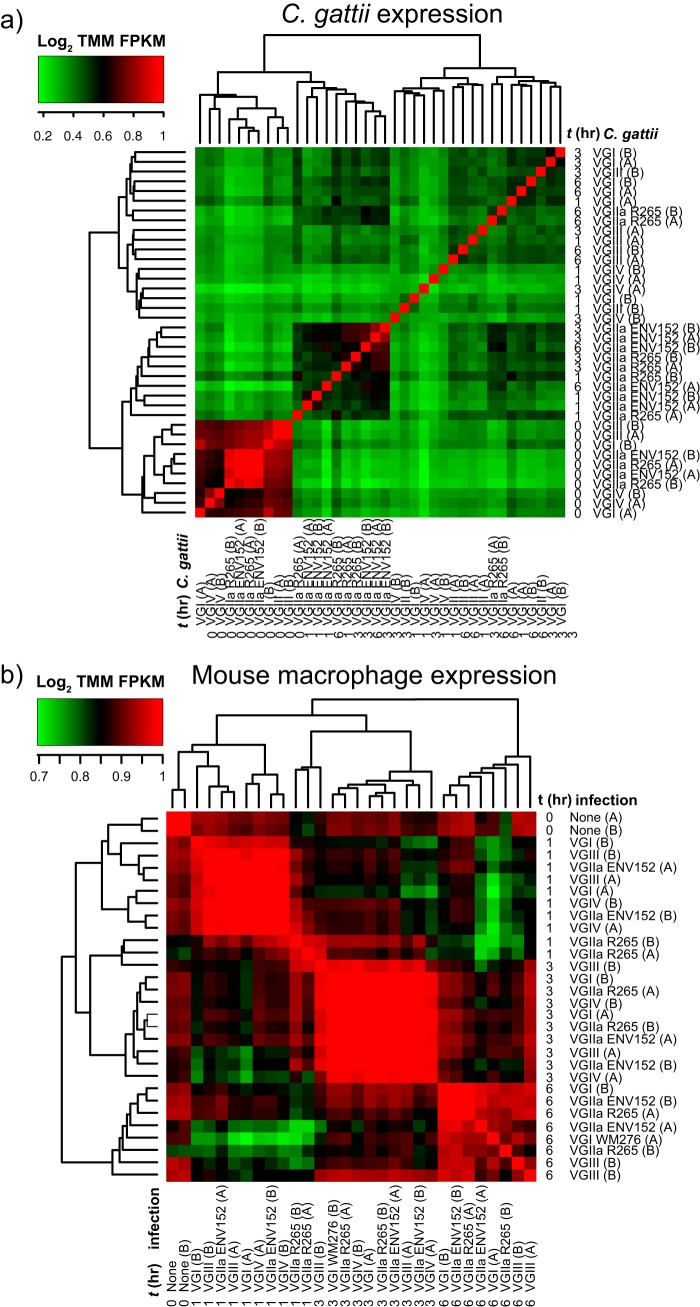
Heat maps of the Log_2_ fold change in the trimmed mean of M-values (TMM) normalized fragments per kilobase of transcript per million mapped reads (FPKM) of C. gattii (a) and mouse BMDM (b) transcripts per sample.

To identify mammalian and Cryptococcus gattii genes that are activated during infection, we performed transcriptome sequencing (RNA-seq) across five C. gattii isolates representing four lineages (VGI, VGII, VGIII, and VGIV), including an environmental and clinical isolate from VGII, both *in vitro* and at time points 1, 3, and 6 h postcoincubation with BMDMs (*ex vivo*). Each of the sequencing runs (*n* = 40) yielded between 904,770 and 17.6 million 30mer reads (10.9 gigabases of total sequence) (see Table S1, tab 1, and Fig. S1, S2, and S3 in the supplemental material), except for VGIV at h 6 (*t*6), which failed to yield sequencing data. Most of the sequences derived from mouse (84% to 99% for each data set), while C. gattii reads ranged from up to 56.97% (*in vitro* without macrophages; see Materials and Methods for mapping description) to between 0.02% and 1.44% reads *ex vivo* (mean of 38,949 reads) (Fig. S2). The C. gattii data sets formed two main clusters: (i) *t*0 (*in vitro*) for all isolates, probably owing to the large difference in read counts between samples, and (ii) VGII isolates at any time point (Fig. 1a). In contrast, gene expression in mouse macrophages clustered by time point (Fig. 1b).

10.1128/mSphere.00445-18.1 FIG S1 Alignments of C. gattii and mouse transcripts to sequences of the core eukaryotic genes (CEGs), suggesting mostly complete C. gattii gene-sets and the high quality of Mouse gene-set mm10 p4. Only VGIIa R265 and mouse mm10p4 were used in this study. Download FIG S1, PDF file, 0.1 MB. Copyright © 2018 Farrer et al. 2018 Farrer et al. This content is distributed under the terms of the Creative Commons Attribution 4.0 International license.

10.1128/mSphere.00445-18.2 FIG S2 RNA was extracted from all four lineages of C. gattii in mouse macrophages at 1 h, 3 h, and 6 h postinfection, as well as C. gattii and macrophages *in vitro* (*t*0). Data represent percentages of sequenced reads deriving from the mouse macrophage (top) and C. gattii (bottom). Download FIG S2, PDF file, 0.2 MB. Copyright © 2018 Farrer et al. 2018 Farrer et al. This content is distributed under the terms of the Creative Commons Attribution 4.0 International license.

10.1128/mSphere.00445-18.3 FIG S3 Heat maps of all differentially expressed genes (FDR *P* value of <0.001 and greater-than-4-fold change of trimmed mean of M-values [TMM] expressed in normalized fragments per kilobase of transcript per million mapped reads [FPKM]) of C. gattii transcripts *in vitro* (*t*0) and during infection of mouse macrophages at 1 h, 3 h, and 6 h. Download FIG S3, PDF file, 0.8 MB. Copyright © 2018 Farrer et al. 2018 Farrer et al. This content is distributed under the terms of the Creative Commons Attribution 4.0 International license.

10.1128/mSphere.00445-18.6 TABLE S1 (Tab 1) Contamination from E. coli K-12 MG1655, suicide vector pCD-RAsl1, and cloning vector pMJ016c identified by BLASTn searches of the nonredundant (NR) database. (Tab 2) Reads aligning either to mouse GRCm38 p4 mm10 gene sets or genome or to R265 updated gene set or genome. ARD, average read depth across genes. Download Table S1, XLSX file, 0.0 MB. Copyright © 2018 Farrer et al. 2018 Farrer et al. This content is distributed under the terms of the Creative Commons Attribution 4.0 International license.

Differential expression was computed using the quantile-adjusted conditional maximum likelihood (qCML) method implemented in EdgeR (17), requiring a false-discovery-rate (FDR) *P* value of <1e^−3^ and at least 4-fold change in trimmed mean of M-values (TMM) normalized fragments per kilobase of transcript per million mapped reads (FPKM) to be considered a significantly differentially expressed gene (DEG). To check for the effect of different read depths on DEG prediction, subsets were created and reanalyzed. This analysis identified consistent numbers of DEGs *in vitro* and more variable numbers between infection time points (Fig. S4).

10.1128/mSphere.00445-18.4 FIG S4 Subsets (75%, 50%, and 25%) of C. gattii data were used to recall differential expression data and for comparisons with the full dataset. *x*-axis categories for each bar chart correspond to data from each of the five isolates (combining *t*1, *t*3, and *t*6) and to *in vitro* data (combining all isolates at *t*0). (a) The total number of differentially expressed gene changes (either up- or downregulation) for isolates *ex vivo* but not *in vitro*. (b) The percentages of genes found in the full dataset (i.e., representing a proxy for true positives). Data are represented as >75% for all categories using 75% subsets, >50% for all categories using 50% subsets, and >25% for all categories using 25% subsets. (c) The number of genes found only in the full dataset (i.e., representing a proxy for false negatives). VGI, VGIII, and VGIV reidentified most of the genes also found with their full datasets, while VGII and *in vitro* conditions recovered fewer genes found in the full dataset as the subset became smaller. (d) The number of genes not found in the full dataset (i.e., representing a proxy for false positives). Values corresponding to previously unidentified genes either increased or decreased as the subset size decreased, with VGIV giving the most robust results and VGIII the least. Download FIG S4, PDF file, 0.2 MB. Copyright © 2018 Farrer et al. 2018 Farrer et al. This content is distributed under the terms of the Creative Commons Attribution 4.0 International license.

### VGII is transcriptionally divergent *in vitro* from other C. gattii lineages.

**FIG 2:**
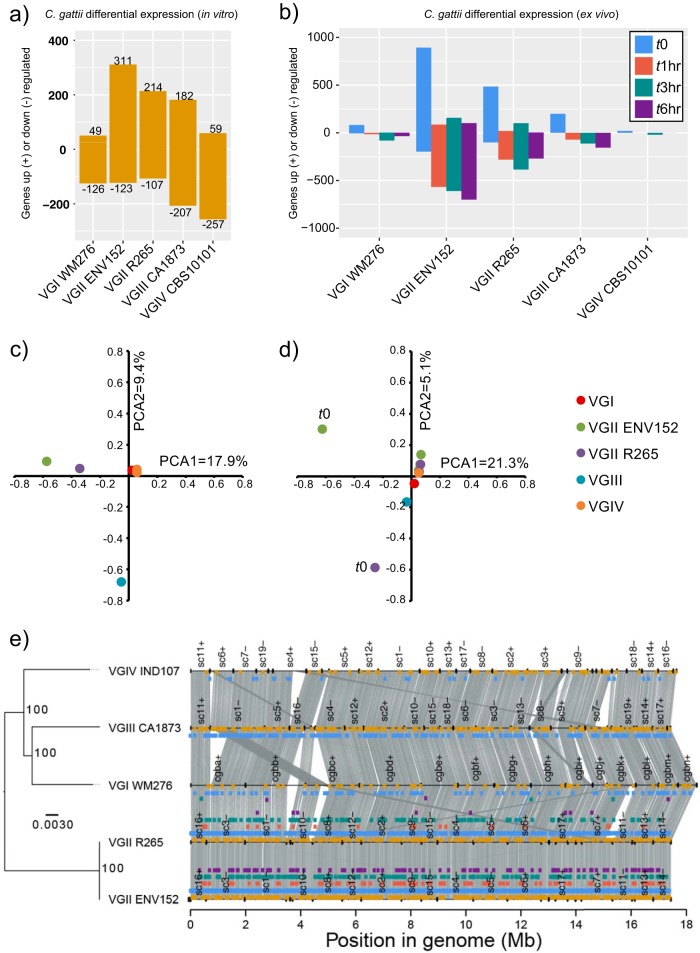
The number of Cryptococcus gattii genes up- and downregulated between lineages *in vitro* (a) and between time points (b) and principal-component analysis (PCA) of differentially expressed genes *in vitro* (c) and between time points (d) and the locations of those genes in their genomes (synteny plotted using Synima [48]) alongside a phylogenetic tree constructed with RAxML (GTRCAT) with 1,000-bootstrap support (shown as asterisks) (e). Genes are considered differentially expressed where the FDR *P* value is <0.001 and the TMM FPKM is greater than 4-fold. Colored boxes show the genomic locations of differentially expressed genes (DEGs) with colors corresponding to panels a and b: *in vitro*, yellow; *t*0 versus another time point, blue; *t*1 versus another time point, red; *t*3 versus another time point, green; *t*6 versus another time point, purple.

Pairwise expression values for all C. gattii isolates *in vitro* (*t*0) revealed 524 DEGs between one or more isolates, indicating that nearly 1 in 10 (8.1%) of all C. gattii protein-coding genes situated throughout the genome (*n* = 6,456) are uniquely differentially regulated among the four divergent lineages, despite being all cultured in the same rich media at 37°C with 5% CO_2_ (Fig. 2a and e) (see also Data Set S1 in the supplemental material). A *G*-test of goodness of fit based on the numbers of *in vitro* DEGs per lineage (not considering specific genes) suggested differences (*G *=* *328.52, X-squared df = 4, *P* value < 2.2e^−16^), and pairwise comparisons using G-tests with Bonferroni multiple correction also showed differences in VGII isolates compared with VGI, VGII ENV152, and VGIV isolates but not VGII R265 (*P* value < 2.2e^−16^). Results of comparisons of VGIV to VGI or VGIII were also highly distinct for numbers of DEGs (*P* value < 2.2e^−16^).

10.1128/mSphere.00445-18.7 DATA SET S1 (Tab 1 to 4) All genes differentially expressed among five C. gattii isolates *in vitro*. (Tab 1) Details of every differentially expressed gene. (Tab 2) Gene annotation, position in genome, and associated PFAM. Orthogroup numbers refer to ortholog groups from 15 C. gattii and C. neoformans H99 genome comparisons (7). (Tab 3) Gene ontology terms assigned to differentially expressed genes. (Tab 4) Differentially expressed genes were found in multiple pairwise comparisons. (Tab 5 to 8) All genes differentially expressed by each of the five C. gattii isolates at different time points *(t*0, *t*3, and *t*6) in coincubation with mouse macrophages. (Tab 5) Details of every differentially expressed gene. (Tab 6) Gene annotation, position in genome, and associated PFAM. Orthogroup numbers refer to ortholog groups from 15 C. gattii and C. neoformans H99 genome comparisons (7). (Tab 7) Gene ontology terms assigned to differentially expressed genes. (Tab 8) Differentially expressed genes were found in multiple pairwise comparisons. (Tab 9 and 10) Expression values and overlap of previously identified differentially expressed genes in Acanthamoeba castellanii and macrophages (13). (Tab 9) C. neoformans genes with similar modulation patterns after interaction of the fungus with amoebae and with murine macrophages. (Tab 10) C. neoformans genes with different modulation patterns after interaction of the fungus with amoebae and with murine macrophages. (Tab 11) Genes differentially expressed by mouse macrophages. LogFc, log fold change; LogCPM, log counts per million. C1, VGIV CBS10101; C2, VGII R265; C3, VGII ENV152; C4, CA1873; C5, VGI WM276. “A” and “B” represent replicates. Download Data Set S1, XLSX file, 2.1 MB. Copyright © 2018 Farrer et al. 2018 Farrer et al. This content is distributed under the terms of the Creative Commons Attribution 4.0 International license.

Of the 524 *in vitro* DEGs, 203 were differentially regulated in both VGII isolates, 245 additional genes were differentially regulated only for an individual VGII isolate, and 50 were differentially regulated inconsistently across VGII (e.g., upregulated in VGIV versus VGII R265 and upregulated in VGI versus both VGII isolates). While some of these genes were also differentially expressed in other lineages, only 5% (*n* = 27) of *in vitro* DEGs were unique to VGI, VGIII, or VGIV isolates, suggesting that VGII harbors expression profiles distinct from those seen with the other lineages. Furthermore, the greater number of genes uniquely differentially expressed within isolates of the VGII isolates suggests that substantial differences exist even within the VGIIa sublineage. Furthermore, VGII and VGIII isolates were distinct from each other by principal-component analysis (PCA) (Fig. 2c), while VGI and VGIV were not distinct from each other.

**FIG 3:**
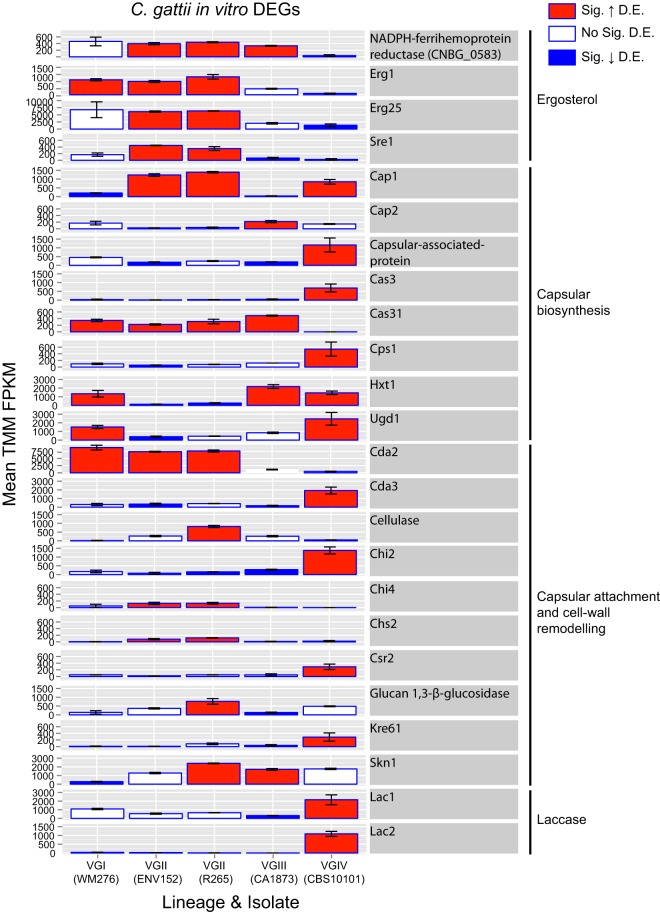
Bar charts showing mean expression (TMM FPKM) of ergosterol, capsular biosynthesis, capsular attachment, and laccase genes differentially expressed between the five isolates of Cryptococcus gattii *in vitro*. Red, significantly (Sig.) upregulated; blue, Sig. downregulated. Genes are considered differentially expressed (D.E.) where the FDR *P* value is <0.001 and the TMM FPKM is greater than 4-fold. Error bars show the range of TMM FPKM values in comparisons between the two replicates.

To understand the role that differential expression may play in each isolate *in vitro*, we opted to use both a targeted approach (looking at known genes of interest [18], including 35 capsule biosynthesis genes, 40 capsule attachment genes and cell-wall remodeling genes, and 20 ergosterol genes based on their orthology to C. neoformans H99 [7]) and a nontargeted approach (gene ontology term [GO-term] and PFAM enrichment). Of the 524 unique DEGs, 8/35 were involved in capsule biosynthesis, 10/40 in capsule attachment and cell-wall remodeling, and 4/20 in ergosterol production, and both laccase genes were differentially expressed in at least one pairwise comparison (Fig. 3). These gene categories are therefore enriched for DEGs based on the results of a hypergeometric test (P[X > x] = *P = *1.207e^−07^).

### *In vitro* C. gattii lineages have distinct expression for capsule biosynthesis and attachment genes.

Capsule biosynthesis genes were differentially expressed between lineages *in vitro* (Fig. 3), including *CAS3*, which was upregulated in VGIV compared with all the other isolates. *CAS3* mutants have a reduced capsule under conditions of combination with *cas31*Δ, *cas32*Δ, or *cas33*Δ mutants and have a partial defect in O-acetylation, leading to reduced overall levels of this modification (18, 19). Meanwhile, the closely related paralog *CAS31* is absent in VGIV and VGIIIb (but present in the VGI, VGII, and VGIIIa lineages) (Fig. S5) and as such has no detectable expression (zero TMM FPKM for both “replicates”)—manifesting as downregulated compared with each of the other lineages. Similarly, *CAP1* is upregulated in VGII and VGIV isolates relative to VGI and VGIII isolates, while the close paralog *CAP2* is upregulated in VGIII compared with VGII. Other differentially expressed capsule biosynthesis genes may not be fully compensated by genetically similar paralogs, such as the hexose transporter *HXT1* downregulated by both VGII isolates.

10.1128/mSphere.00445-18.5 FIG S5 A paralogous cluster of Cas3 and Cas31 from a previously described study of 16 isolates (7) had their sequences aligned using MUSCLE v3.8.31 (46), and a neighbor-joining tree was constructed using PAUP version 4.0b10 (47) to decipher orthologs. *Cn*, Cryptococcus neoformans; *Cg*, Cryptococcus gattii. Note that VGIV has no Cas31 gene. Expression levels (TMM FPKM) for five isolates are shown below the dendrogram. Download FIG S5, PDF file, 0.2 MB. Copyright © 2018 Farrer et al. 2018 Farrer et al. This content is distributed under the terms of the Creative Commons Attribution 4.0 International license.

Capsule attachment and cell-wall remodeling genes were also differentially expressed between lineages of C. gattii (Fig. 3). For example, chitin synthases *CHI4* and *CHS2* were upregulated in both VGII isolates. Chitinase *CHI2* was upregulated in VGIV. Chitin deacetylase 1 (*CDA1*) was highly expressed by all isolates *in vitro* (TMM FPKM ranging from 932 to 2,265 across all replicates) and was not identified as differentially expressed. However, *CDA2* was upregulated in VGI and VGII isolates compared with VGIII and VGIV. Meanwhile, *CDA3* was upregulated in VGIV compared with VGII and VGIII (perhaps again showing a change in paralog expression regulation). Both *CDA2* and *CDA3* mutants have increased capsule size when combined with *cda1*Δ mutants (18), however, *CDA1* was not found to be differentially expressed.

### Differential expression of known drug targets and virulence genes.

In common with most fungi, ergosterol is found in the membrane of C. gattii and is a key target for numerous antifungal drugs including fluconazole and amphotericin B (20). The VGII lineage had significantly higher expression for several genes involved in ergosterol production, including NADPH-ferrihemoprotein reductase genes, *ERG1* (squalene monooxygenase), *ERG25* (methylsterol monooxygenase), and *SRE1*. Each of these genes are linked to drug resistance or the oxidative stress response; for example, *ERG1* mutants have increased fluconazole susceptibility in Candida glabrata (21), *ERG25* has a moderate susceptibility to hypoxia and the endoplasmic reticulum (ER) stress-inducing agent dithiothreitol (DTT) in Aspergillus fumigatus (22, 23), and *SRE1* is required for hypoxic induction of genes coding for oxygen-dependent enzymes involved in ergosterol synthesis in C. neoformans (24).

Laccases are cell wall enzymes that catalyze melanin to protect *Cryptococcus* from various stresses, including oxidative stress, and are therefore considered important virulence factors (25). Laccase production in both C. gattii and C. neoformans is controlled by two cell wall enzymes that control melanin production (25). VGIV significantly upregulates both copies (*LAC1* and *LAC2*) *in vitro* compared with VGIII and all the other isolates, respectively. Whereas *LAC1* and *LAC2* flank each other in C. neoformans, C. gattii has an additional gene (CNBG_2145) encoding a hypothetical protein with no functional annotation (PFAM, GO, KEGG) in the middle. This gene, unlike *LAC1* and *LAC2*, was not consistently expressed by any of the lineages under any conditions.

Differential expression levels among isolates *in vitro* were also enriched (two-tailed Fisher’s exact test with false-discovery-rate [FDR {*q*-value}] analysis) for several GO-terms compared with the remaining genes (Table 1). Enriched terms included “oxidative reduction” (*q *=* *1.85E^−07^) and oxidoreductase activity (*q *=* *0.011). Genes with the oxidative reduction GO-term, including those coding for ferric reductases, metalloreductases, nitric oxide dioxygenases, acidic laccases, oxidoreductases, and various dehydrogenases, were both up- and downregulated by each isolate. VGII isolates had the greatest number of upregulated genes assigned an oxidative reduction function: VGII ENV152 (*n* = 39), VGII R265 (*n* = 23), compared with VGIII (*n* = 18), VGIV (*n* = 11), and VGI (*n* = 7). All R265 genes, apart from the single gene CNBG_2804 (encoding a hypothetical protein with a DUF455 domain), were also found in ENV152. The remaining 17 uniquely upregulated genes in ENV152 included genes encoding five dehydrogenases (methylmalonate-semialdehyde, 3-hydroxyacyl-coenzyme A [hydroxyacyl-CoA], glutaryl-CoA, glyceraldehyde-3-phosphate, and glutamate), the Fe-Mn family superoxide dismutase, and ferric reductase transmembrane component 4. Only a single PFAM, a PF00083.19 sugar (and other molecule) transporter, was found to be enriched in the *in vitro* comparisons. The ability of C. gattii to respond to host- and environment-derived oxidative stress is well described (22, 26, 27), and it is noteworthy that differences in expression levels between isolates and lineages were found even under *in vitro* conditions.

**TABLE 1:** Enrichment of functional annotation for *in vitro* and *ex vivo* comparisons^*a*^

GO/PFAM designation	Isolate	Count 1	Count 2	Fisher *P*	*q* value	Rel. prop	GO description
Sig. enriched terms from *in vitro* comparisons							
GO:0055114		64	256	1.13E−10	1.85E−07	2.54	Oxidation reduction
GO:0016638		5	2	1.01E−04	1.11E−02	25.41	Oxidoreductase activity, acting on the CH-NH2 group of donors
GO:0050660		13	32	9.79E−05	1.11E−02	4.13	FAD binding
PF00083.19		18	54	2.26E−05	2.66E−02	3.56	Sugar (and other) transporter
GO:0004553		12	33	4.15E−04	3.25E−02	3.7	Hydrolase activity, hydrolyzing O-glycosyl compounds
GO:0005975		25	116	7.41E−04	4.86E−02	2.19	Carbohydrate metabolic process
							
Sig. depleted terms from *in vitro* comparisons							
GO:0043234		3	211	3.35E−06	6.14E−04	0.14	Protein complex
GO:0034645		11	316	6.33E−05	9.42E−03	0.35	Cellular macromolecule biosynthetic process
GO:0006396		2	142	2.60E−04	2.25E−02	0.14	RNA processing
GO:0003723		2	137	3.78E−04	3.11E−02	0.15	RNA binding
GO:0005737		35	597	7.23E−04	4.86E−02	0.6	Cytoplasm
GO:0015031		2	126	7.96E−04	4.86E−02	0.16	Protein transport
							
Sig. enriched terms from *ex vivo* comparisons							
GO:0055114	VGI WM276	18	302		3.56E−05	4.63	Oxidation reduction
GO:0016491	VGI WM276	18	376		4.52E−04	3.72	Oxidoreductase activity
GO:0006364	VGII ENV152	34	13		1.93E−12	10.64	rRNA processing
GO:0005730	VGII ENV152	25	6		7.74E−11	16.95	Nucleolus
GO:0003735	VGII ENV152	54	58		7.37E−10	3.79	Structural constituent of ribosome
GO:0006412	VGII ENV152	71	117		4.27E−07	2.47	Translation
GO:0003723	VGII ENV152	51	88		1.32E−04	2.36	RNA binding
GO:0005506	VGII ENV152	35	51		3.66E−04	2.79	Iron ion binding
GO:0055114	VGII ENV152	95	225		5.04E−04	1.72	Oxidation reduction
GO:0016627	VGII ENV152	13	8		1.32E−03	6.61	Oxidoreductase activity, on CH-CH
GO:0008026	VGII ENV152	24	32		2.60E−03	3.05	ATP-dependent helicase activity
GO:0016627	VGII R265	11	10		4.25E−04	9.6	Oxidoreductase activity, on CH-CH
GO:0022900	VGII R265	8	7		8.59E−03	9.97	Electron transport chain
GO:0020037	VGII R265	11	17		8.59E−03	5.64	Heme binding
GO:0016491	VGIII CA1873	33	361		9.41E−04	2.57	Oxidoreductase activity
GO:0055114	VGIII CA1874	28	292		1.77E−03	2.69	Oxidation reduction
							
Sig. depleted terms from *ex vivo* comparisons							
GO:0043234	VGII ENV152	20	194		1.39E−03	0.42	Protein complex
GO:0006508	VGII ENV152	12	132		9.48E−03	0.37	Proteolysis

a Enrichment of functional annotation for *in vitro* and *ex vivo* comparisons was determined by a two-tailed Fisher exact test with a *q*-value (Storey-Tibshirani) FDR, where count 1 data represent numbers of terms and parent terms in the differentially expressed set and count 2 data represent the remaining terms and parent terms in the non-differentially expressed set. The uncorrected Fisher *P* values, corrected *q* values, relative-proportion (Rel. Prop) values, and descriptions are provided for each term (requiring *q* values of <0.05 for each). FAD, flavin adenine dinucleotide; Sig., significantly.

### One in five C. gattii genes is differentially expressed under *in vitro* and *ex vivo* conditions among the lineages.

Pairwise comparisons of expression values for each lineage at different time points (*t*0 versus *t*1, *t*3, and *t*6) identified 1,193 unique DEGs, the majority of which were upregulated *in vitro* versus *ex vivo* (Fig. 2b) (see also Data Set S1, tab 2). About 1/3 (60%) (*n* = 309) of these genes were also differentially expressed in an interlineage *in vitro* comparison, leaving 215 genes that were uniquely differentially expressed *in vitro*. PCA showed large differences between *t*0 and *ex vivo* time points in both VGII isolates (Fig. 2d).

**FIG 4:**
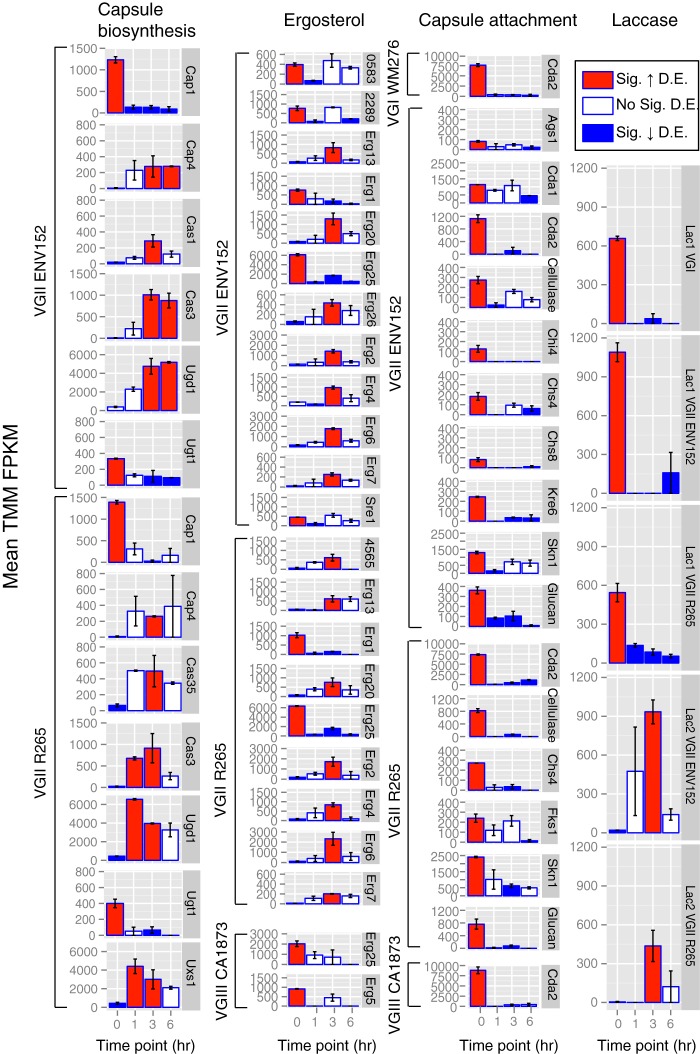
Bar charts showing mean expression (TMM FPKM) of differentially expressed ergosterol, capsular biosynthesis, capsular attachment and laccase genes between the five isolates of Cryptococcus gattii at each time point (*t*0 = *in vitro*, *t*1 = 1-h w/BMDMs, *t*3 = 3 h w/BMDMs, and *t*6 = 6 h w/BMDMs). Red, significantly upregulated; blue, significantly downregulated. Genes are considered differentially expressed where FDR *P* value < 0.001 and greater than 4-fold TMM FPKM. Error bars show the range of TMM FPKM values in comparisons between the two replicates. Glucan, glucan 1,3-β-glucosidase.

Genes of interest (capsule, ergosterol, and laccases) were enriched for DEGs at different time points based on the results of a hypergeometric test (P[X > x]) = *P = *0.007), including nine capsule biosynthesis genes (all VGII), 11 capsule attachment and cell-wall remodeling genes (all downregulated *ex vivo*), 14 ergosterol genes, and both laccase genes (Fig. 4). Separately, many (*n* = 40/97; 41%) C. neoformans genes that were previously found to be differentially expressed via microarray in the presence of amoebae and macrophages (13) were similarly modulated in C. gattii (111 genes with similar or different modulation results between amoebae and macrophages, 97 of which had a C. gattii ortholog, 56 of which were also C. gattii DEGs between time points, and 40 of which had the same directionality) (Data Set S1, tab 3).

Genes differentially expressed *ex vivo* were statistically significantly enriched (two-tailed Fisher’s exact test with *q*-value FDR) for 18 GO-terms and no PFAM terms (Table 1). Strikingly, the oxidoreductase activity term was enriched in each lineage and isolate (apart from VGIV, which had only 20 differentially expressed genes and thus no enriched terms). The ability of C. gattii to respond to host- and environment-derived oxidative stress is thus a significant feature of genes that are differentially expressed (both between lineages *in vitro* and under *in vitro* versus *ex vivo* conditions). Additional terms included iron ion binding and terms related to ribosomes (i.e., processing of rRNA, a structural constituent of ribosome) for VGII ENV152, perhaps indicating an increase in translational activity.

Capsule biosynthesis genes in VGII were differentially expressed in the presence of macrophages (Fig. 4). For example, *CAP1* and *UGT1* were downregulated by both VGII isolates, while *CAP4*, *CAS3*, and *UGD1* were all upregulated at one or more of the *ex vivo* time points. Other capsule biosynthesis DEGs included *CAS35* (the *CAS35*Δ mutant has a decreased capsule [19]), which was upregulated in R265 at *t*3 versus *t*0; *UXS1* (the *uxs1*Δ capsule is missing xylose [28]), which was upregulated in R265 at *t*1 and *t*3 versus *t*0; and *CAS1* (the *cas1*Δ mutant has a defect in capsule O-acetylation and reactivity to GXM antibodies [29]), which was upregulated in VGII ENV152 at *t*3.

Capsule attachment and cell-wall remodeling genes were also differentially expressed *ex vivo*, predominantly by VGII isolates, and were all downregulated *ex vivo* (Fig. 4). For example, chitin synthase genes *CHS4* and *CHS8* were upregulated *in vitro* in VGII compared with any other time points *ex vivo*. Chitins generated by such chitin synthases are converted into chitosan by the chitin deacetylase genes *CDA1*, *CDA2*, and *CDA3*, where it constitutes an important component of the cell wall of *Cryptococcus* (30). Both *CDA1* and *CDA2* are downregulated *ex vivo* in one or more of the lineages of C. gattii, apart from VGIV.

The four ergosterol genes (CNBG0583/CNAG01003, *ERF1*, *SRE1*, and *ERG25*) that were upregulated in VGII compared with VGIII and VGIV *in vitro* were also upregulated in VGII *in vitro* compared with the three *ex vivo* time points, again suggesting that these are downregulated during infection. However, nine additional ergosterol biosynthesis genes, including *ERG2*, *ERG6*, *ERG7*, *ERG13*, *ERG20*, and *ERG26*, were each upregulated in the VGII isolates at 3 h postinfection compared with the levels seen *in vitro*, suggesting that these genes are activated between 1 and 6 h after coincubation with macrophages.

The laccase genes that produce melanin and that are upregulated in VGIV *in vitro* compared with the other lineages are also differentially expressed in VGII between *in vitro* and *ex vivo* conditions. Specifically, *LAC1* (CNBG2144) in VGI and VGII is downregulated *ex vivo*. In contrast, *LAC2* (CNB2146) is upregulated in both VGII isolates at *t*3 compared with *t*0—demonstrating that during infection, VGII isolates switch expression from *LAC1* to *LAC2*.

### Mouse macrophage response to C. gattii.

**FIG 5:**
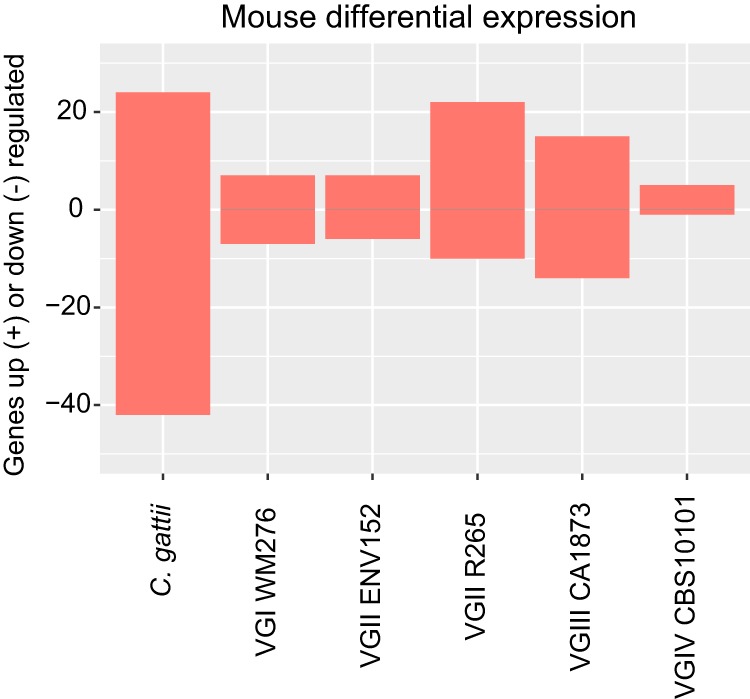
A bar chart showing the number of mouse genes up- and downregulated by each of the five isolates of C. gattii compared with growth without yeast. Where the same gene was found in multiple pairwise comparisons, it is included only once in the redundant categories (applicable only for the grouped category). Positive values indicate genes that were upregulated in that isolate/lineage compared with others, while negative values (below zero) indicate genes that were downregulated in that isolate/lineage compared with others. Genes are considered differentially expressed where FDR *P* value are <0.001 and TMM FPKM values are greater than 4-fold.

Mouse BMDM expression for each of 58,716 annotated mouse transcripts (including protein-coding genes and pseudogenes and other non-protein-coding genes) was highly consistent under *in vitro* conditions and conditions of coincubation with any of the four lineages of C. gattii at any of the three time points (Fig. 5), perhaps indicating low rates of yeast engulfment or the general low immunogenicity of cryptococci. In this case, differential expression of C. gattii *ex vivo* (see previous section) may be caused by indirect effects of macrophage coincubation. Only 24 upregulated DEGs and 42 downregulated DEGs were identified in total (Data Set S1, tab 4). Of those 24 upregulated genes, five separate/unique FBJ osteosarcoma viral oncogenes (specifically, *FOSB* and the truncated splice form Δ*fosb2*) belonging to the eight most highly differentially expressed genes (log fold change = 3.58 to 7.78) were found at *t*1 for VGII R265 only. *FOS* genes encode a leucine zipper protein that dimerizes with the Jun family (among which JunB is also upregulated at *t*3 in VGII ENV152), forming the AP-1 transcription factor, which in turn regulates diverse functions, including cell proliferation, differentiation, and transformation, following the primary growth factor response. Perhaps it is therefore unsurprising that 20% of upregulated genes (*n* = 11/52 redundant upregulated genes), including *EGR1-201* and *EGR1-202*, at *t*3 for all five C. gattii isolates tested and *EGR3* at *t*1 for VGII R265 only, belonged to the early growth response protein 1 family genes.

Most of the genes downregulated in mouse macrophages during infection appear to be signaling molecules and transcription factors, including ETV5 (ETS transcription factor variant 5), which is downregulated at *t*6 in all C. gattii isolates except VGIV. Other downregulated genes include those encoding the DENND2C proteins at *t*3 in VGII ENV152 and at *t*1 in VGII R265. DENND2C proteins act in diverse intracellular signaling pathways via GDP/GTP exchange, with many potential downstream targets. Additionally, DNA-binding protein ID1 (which inhibits basic helix-loop-helix [bHLH] transcription factors) was downregulated at *t*6 in VGI, VGII ENV152, and VGIII, as well as *t*1 in VGII R265. Similarly, dual specific protein phosphatase 6 (DUSP6), involved in mitogen-activated protein kinase (MAPK) signaling, was downregulated at *t*6 in VGI, VGII ENV152, and VGIII, while DUSP4 was also downregulated at *t*6 in VGI only.

## DISCUSSION

The transcriptional responses of host and pathogen during infection can reveal key insights into their interactions and the molecular basis of pathogenicity. Transcriptional differences between distantly related isolates in culture can also reveal the impact of their genetic divergence and explain epidemiological differences. In this study, we compared the expression profiles of five isolates belonging to the four lineages of C. gattii *in vitro* between three early time points during coincubation with mouse bone marrow-derived macrophages (BMDM) and, in parallel, characterized the host response. Both C. gattii comparisons suggested that lineage VGII is transcriptionally divergent from non-VGII lineages in terms of number of genes differentially expressed. For example, only 5% of *in vitro* DEGs belonged to non-VGII isolates, despite these isolates accounting for 30% of all pairwise comparisons made. It is likely that the loss of RNA interference (RNAi) functionality in VGII (7, 31, 32) is partially responsible indirectly (rewiring of gene regulatory responses in result to loss of Argonaut proteins) and perhaps even directly (mRNA not being degraded).

VGII is responsible for nearly all C. gattii infections in the Pacific Northwest (PNW) (33), which is the location of the worst recorded outbreak of C. gattii infections worldwide. In this study, we found that two subgroup VGIIa isolates had distinct expression profiles, possibly owing to genetic variation generated from their different sources of origin (environmental or clinical). VGII isolates also show a high degree (albeit across a range) of virulence traits, including rate of intracellular proliferation within macrophages (R265 = 1.8 ± 0.1 and ENV152 = 2.3 ± 0.2 compared with VGI WM276 = 0.98 ± 0.2 and VGIV CBS10101= 1.23 ± 0.3), average mitochondrial tubularization percentage (R265 = 58.4% and ENV152 = 44.5% compared with VGI WM276 = 14.3% and VGIV CBS10101 = 21.6%), average phagocytosis percentage (R265 = 31.4% and ENV152 = 20.5% compared with VGI WM276 = 11.1% and VGIV CBS10101 = 8.6%), and macrophage cell death percentage (R265 = 15.2% and ENV152 = 12.3% compared with VGI WM276 = 16.8% and VGIV CBS10101 = 12%) (34, 35). VGIII isolate CA1873 was not evaluated for these phenotypic traits in those two papers. Therefore, isolates within lineages demonstrate high phenotypic variability. Although the sequenced isolates in this study are representative of each lineage, comparing multiple isolates from each lineage would help delineate the intralineage versus interlineage variations in gene expression.

The capsule biosynthesis pathway in C. gattii constitutes a complex trait controlled by numerous genes. Many of these genes presented various degrees of expression among the four lineages *in vitro*, suggesting the presence of diverse mechanisms operating to maintain and perhaps even diversify the properties of the capsule. However, non-VGII lineages did not differentially express capsule synthesis genes *ex vivo*, suggesting that they are perhaps less rigorously regulated (expressed under more diverse conditions) or expressed less abundantly or are perhaps less sensitive to host-derived stresses and stimuli. One such gene, *CAS3*, is upregulated by VGII isolates at multiple time points *ex vivo* compared with *in vitro*. *CAS3* has previously been identified as upregulated in VGII R265 compared with low-virulence VGII isolate R272 under conditions of carbon and nitrogen starvation (16). However, we found no evidence of differential expression of *CAS3* between VGII R265 and VGII ENV152—possibly indicating a uniqueness of R272 expression or experimental differences between studies. However, we found that *CAS3* is upregulated by VGIV CBS10101 *in vitro* compared with other isolates, while close paralog *CAS31* is a lineage-specific gene missing in VGIV (7). It is therefore possible that VGIV is overexpressing *CAS3* to compensate for its *CAS31* deletion or disruption and demonstrating subfunctionalization of these paralogs. Furthermore, *cas31*Δ mutants have previously been shown to manifest minor differences in GXM composition in C. neoformans (18, 19), which may also manifest in C. gattii VGIV as well as in VGIIIb. Separately, the capsule biosynthesis gene *CAP2* is upregulated by VGIII, while *CAP1* is downregulated by VGI and VGIII, suggesting a lineage transition from expressing one gene to expressing another. Capsule attachment DEGs (in comparison to capsule biosynthesis genes) were all downregulated *ex vivo*, suggesting that these do not play an active role during infection.

Ergosterol in the membrane of C. gattii is a key target for numerous antifungal drugs, including fluconazole and amphotericin B (20). VGII presented higher expression for genes involved in ergosterol production *in vitro*, including upregulation of *ERG1*, *ERG25*, *SRE1*, and an NADPH-ferrihemoprotein reductase gene. Mutants for each of these genes show a range of defects in the presence of antifungals or hypoxia (21–24). *ERG1*, *ERG25*, and *SRE1* were also upregulated in VGII *in vitro* compared with the three *ex vivo* time points, suggesting that these genes can be switched off during non-drug-related stresses. However, a further nine ergosterol genes were upregulated between 1 and 6 h postinfection, including *ERG2*, *ERG6*, *ERG7*, *ERG13*, *ERG20*, and *ERG26*, suggesting that this pathway is active during infection. The biological significance of these expression differences are unclear but could manifest in lineage-specific drug resistance variations.

Laccase production in both C. gattii and C. neoformans is controlled by two cell wall enzymes (encoded by *LAC1* and *LAC2*) that possess a broad spectrum of activity, oxidizing both polyphenolic compounds and iron (25). VGIV upregulates both genes compared with other lineages *in vitro*. *LAC1* is downregulated in VGI and VGII *ex vivo* compared with *in vitro*, and *LAC2* is instead upregulated in both VGII isolates at *t*3 compared with *t*0—suggesting that C. gattii VGII switches expression from one laccase gene to the other during infection, perhaps due to changing concentrations or requirements for metabolism of lactose and galactose or for production of melanin. In C. neoformans, *LAC1* and *LAC2* (along with capsule genes) are part of the Gpa1-cAMP pathway, which regulates capsule and melanin production using l-3,4-dihydroxyphenylalanine (l-dopa) as a substrate (36). We hypothesize that VGII uses *LAC2* to regulate growth and glucose responses (and potentially virulence) instead of *LAC1*. In C. neoformans, *LAC1* is localized to the cell wall, whereas *LAC2* is cytoplasmic but is capable of localizing to the cell wall (37); therefore, C. gattii *LAC2* could behave similarly to its C. neoformans counterpart, which may have increased versatility during infection of macrophages.

We also identified differential expression levels in VGII lineage-specific genes such as those encoding an MFS transporter and an oxidoreductase that may have contributed to the enrichment of oxidative reduction, which was identified in both *in vitro* comparisons and *in vitro* versus *ex vivo* comparisons. This term includes a wide range of functions, pathways, and genes, including those encoding ferric reductases, metalloreductases, nitric oxide dioxygenases, acidic laccases, oxidoreductases, and various dehydrogenases. Previously, an upregulation of genes involved in oxidative stress has been identified in C. neoformans isolate H99 coincubated with the J774A macrophage-like cell line for 16 h versus *in vitro* conditions (12) and in C. gattii VGII isolate R265 versus isolate R272 under carbon and nitrogen starvation conditions (16). The ability of C. gattii to respond to host- and environment-derived oxidative stress is well described—and it is consistent that genes involved in these processes should also be enriched between isolates and lineages at the expression level *in vitro* and among lineages at different time points *ex vivo*.

Finally, we found that mouse macrophages respond to C. gattii by upregulating FosB/Jun/Egr1 regulatory proteins at early time points, which may trigger differentiation and cell division of macrophages. We found little evidence for differential expression induced by different C. gattii lineages, suggesting that the macrophage responses to C. gattii are the same across lineages. Our report highlights the breadth of expression profiles among the lineages of C. gattii and the diversity of transcriptional responses at this host-pathogen interface, some of which may be the cause of the differences in phenotypic and clinical manifestations noted between lineages (38).

## MATERIALS AND METHODS

### Macrophage and Cryptococcus gattii infection assay.

C. gattii was grown in accordance with methods described in previous work (26). Briefly, five strains of C. gattii were grown in RPMI/C10 media with heat-inactivated (HI) fetal bovine serum (FBS) media and were preincubated at 37°C in C10 media for 1.5 h before infection. Bone marrow-derived macrophages (BMDM) were derived from bone marrow cells collected from the femur and tibia of C57BL/6 female mice. All mouse work was performed in accordance with the Institutional Animal Care and Use Committees (IACUC) guidelines and with relevant guidelines at the Broad Institute and Massachusetts Institute of Technology, with protocol 0615-058-1. Primary bone marrow cells were grown in C10 media as previously described (39) and supplemented with macrophage colony-stimulating factor (M-CSF) (Thermo Fisher Scientific) at final concentration of 10 ng/ml to promote differentiation into macrophages.

For the infection experiment, dilutions of 7.5E+5 cells were made for BMDM and C. gattii strains. BMDM were spun at 500 relative centrifugal force (rcf) at 37°C for 2 min to adhere to plates, and inoculated with C. gattii strains using a multiplicity of infection (MOI) of 1 macrophage to 2 C. gattii. Macrophages with C. gattii cells were centrifuged at 500rcf at 37°C for 2 min. Next, cells were incubated at 37°C with 5% CO_2_ for 1 h, 3 h, and 6 h postcentrifugation. In addition, we grew C. gattii *in vitro*, and BMDM without C. gattii, in duplicate with the same C10 media. At the end of the time points, the medium was removed, lysis buffer was added, and samples were placed in a −80°C freezer for RNA extraction.

RNA was extracted from population samples using a Qiagen RNeasy minikit. All samples were subjected to 3 min of bead beating with 0.5-mm-diameter zirconia glass beads (BioSpec Products) in a bead mill. Libraries were generated usinga TruSeq Stranded mRNA Library Prep kit (Illumina).

### RNA-seq and data analysis.

All samples were sequenced on an Illumina HiSeq 2500 system to generate strand-specific paired-end reads that were 38 nucleotides (nt) in length. Raw reads from each sample were sequenced on multiple lanes and so were merged into individual replicates. Using BLAST searches of the nr database, we identified the following contaminants in each of the samples: Escherichia coli K-12 MG1655, suicide vector pCD-RAsl1, and cloning vector pMJ016c (see Table S1, tab 1, in the supplemental material). These contaminants were excluded from all of the read sets on the basis of Bowtie2 (40) alignments. We next aligned all reads to mouse GRCm38 p4 mm10 transcript sets (including protein-coding genes and pseudogenes and other categories of non-protein-coding genes) and genomes (41) using Bowtie2 (40). We took all reads that aligned to neither the mouse genome nor the gene sets and aligned those reads to the R265 updated gene set (7) as well as the R265 genome using Bowtie2 (Table S1, tab 2). Bowtie2 alignments to mouse or C. gattii were run though the Trinity version r20140413p1 (42) pipeline, using the following default parameters: max insert size 800, no-mixed (no unpaired reads), no-discordant (does not satisfy the paired-end constraints), gbar = 1,000 (disallow gaps within 1,000 bases), and end-to-end (entire read must align from one end to other without any trimming or soft clipping). To reveal the extent of the effect that this had on alignment data, we aligned R265 RNA *in vitro* replicate A to the genome of R265 with Bowtie2 using nonstringent parameters. The overall alignment rate increased from 56.97% to 68.62%. We also found that some reads that were left unaligned by Bowtie2 were aligned by the separate tool BWA v0.7.4.

Other factors that were likely to reduce alignment coverage were the draft quality of the C. gattii R265 genome and the gene sets used (see Fig. S1 in the supplemental material for evaluation of Core Eukaroyotic Gene coverage)—as a consequence of the effects of those limiting factors, genuine C. gattii mRNA would be unalignable. Another factor was that non-VGII isolates were aligned to VGII and that certain differences, including lineage-specific gene differences, would reduce gene coverage. However, the alignment strategy employed was deemed more suitable than aligning to individual lineage reference sets and relying on orthologs, which would reduce the number of genes subjected to comparisons and add errors caused by incorrect assignment of orthologs.

All reads from *in vitro* C. gattii experiments that were unaligned were assembled *de novo* using Trinity (5,730 sequences of total length 2.1 Mb), genes predicted by Transdecoder (521 sequences of 270 kb), and BLASTn searches of the NCBI nr database. Only 90 of these sequences (37 kb) did not align to either the mouse genome or that of C. gattii R265. The remainder of the unaligned sequence that did not map to mouse or C. gattii was highly repetitive and was enriched for Illumina adapter and control sequences. Indeed, FASTQC v0.11.4 identified large fractions of the unaligned unprocessed reads derived from Process Controls for TruSeq kits, which including >10% CTA and CTL as overrepresented sequences.

Reads that aligned only to coding sequence (CDS) from C. gattii or mm10 had their transcript abundances estimated using RSEM (43) and differential expression levels (FDR *P* value < 0.001 and >4-fold change of TMM-normalized FPKM) predicted using EdgeR (17) through the use of the Trinity version r20140413p1 (46) pipeline. Specifically, the pipeline uses the EdgeR quantile-adjusted conditional maximum likelihood (qCML) method after estimating common dispersion and tagwise dispersions using the Cox-Reid profile-adjusted likelihood method (also implemented in EdgeR). For *Cryptococcus* strains, we estimated transcript abundance and differential expression in the same way after (i) merged time points (i.e., calculations performed for each isolate only) and (ii) merged isolates (i.e., calculations performed for each time point only). Merging of isolate data resulted in far weaker correlations between replicates than merging of time point data, indicating that *in vivo* conditions were more influential on overall expression values than lineage/strain-specific differences. Principal-component analysis (PCA) was performed using SmartPCA from EIGENSOFT v4.0 (44), where each gene was given a value of 0 for non-DEG and a value of 1 for DEG (upregulated).

Orthologs to genes of known function in C. neoformans H99 were identified in C. gattii R265 using OrthoMCL (45) across 16 isolates as previously described (7). Genes of interest (capsule biosynthesis, capsule attachment, and ergosterol genes) that fell within paralogous clusters had the sequences of 16 isolates described previously (7) aligned using MUSCLE v3.8.31 (46), and a neighbor-joining tree was constructed using PAUP version 4.0b10 (47) to decipher orthologs. Synteny was visualized using Synima (48).

Of the 58,716 annotated mouse transcripts, 39,229 (67%) had evidence of expression in one or more samples (TMM FPKM > 1). Prior to differential expression analysis, we excluded 3,596 mouse transcripts that any C. gattii *in vitro* RNA (i.e., no macrophage present) aligned to them. Surprisingly, some mouse transcripts were very highly expressed in C. gattii-only samples (e.g., glutamate receptor interacting protein 2 [Grip2-205] had a TMM FPKM value of >15,000 for every C. gattii isolate and values between only 10 and 874 for *t*1, *t*3, and *t*6, which were time points when mouse macrophages were actually present), perhaps owing to sequence similarity of some 30mers or to inaccuracies in the mouse gene set. Applying the same threshold FDR *P* value of <0.001 and >4-fold change of TMM normalized FPKM using EdgeR (17) identified 24 genes that were upregulated during infection by 1 or more isolates of C. gattii and 42 genes that were downregulated during infection by 1 or more isolates of C. gattii.

Enrichment analyses for PFAM and GO terms previously assigned (7) were conducted using two-tailed Fisher’s exact test with *q*-value FDR. Multiple testing corrections were performed with the Storey-Tibshirani (49) method (requiring a *q* value of <0.05). For enrichment tests, we excluded PFAM and GO terms related to transposable elements and domains of unknown function.

### Impact of read depth on differential expression.

On the basis of data quantity/transcriptome coverage alone, the host response and *in vitro* C. gattii lineage expression differences should be the most robust, while C. gattii *ex vivo* expression changes should be less robust. To explore the coverage and sensitivity of our C. gattii RNA-seq data and pipeline, we first made subsets (75%, 50%, and 25%) of our C. gattii data and recalibrated the differential expression data (Fig. S3). The number of genes differentially expressed between isolates under *in vitro* conditions remained most consistent in terms of number of differentially expressed genes following subsetting (100% = 1,208 genes, 75% = 1,205, 50% = 1,147, 25% = 800) (Fig. S3A).

For all C. gattii data sets (those corresponding to *in vitro* conditions and individual isolates corresponding to comparisons between conditions), the proportion of genes reidentified in the 75% subset was between 79% to 100% (VGIV CBS10101 had the same 20 genes corresponding to both data set sizes) (Fig. S3B). In contrast, the number of genes identified only in the full data sets increased in the remaining subsets, with the most pronounced increase being that seen with VGIIa ENV152, which lost the identification of 476 genes in the 75% subset, possibly indicating a lack of data for this isolate (Fig. S3C). We also assessed each subset for genes that were not found in the full set (i.e., genes that were unique to the subsets; Fig. S3D). VGII and VGIV and the *in vitro* data sets had fewer unique genes as the subsets became smaller, while VGI and VGIII had increases as the subsets became smaller.

Under *in vitro* conditions, between 10% to 15% of genes were unique in their subsets, while for VGII ENV152, the proportion was between 1% and 5%. For isolates with very few identified differentially expressed genes (such as VGIV CBS10101 and VGIII CA1873), the numbers of unique genes approached or even exceeded the number found in the full data set. Given this variation, it is therefore likely that most of the *ex vivo* C. gattii data sets would benefit from deeper RNA-seq data and that the *in vitro* expression values are more robust than the *ex vivo* data. Nevertheless, the majority of differential expressed genes were consistent between the larger subsets of data.

### Accession number(s).

All RNA-seq data for mouse macrophages and C. gattii have been deposited in the Short Read Achieve under accession no. PRJNA428946.
