# Supplementary figures and images for: Transcriptional Heterogeneity of Cryptococcus gattii VGII Compared with Non-VGII Lineages Underpins Key Pathogenicity Pathways
Source: mSphere. 2018 Oct 24;3(5):e00445-18. doi: 10.1128/mSphere.00445-18 (PMC6200987; doi:10.1128/mSphere.00445-18)

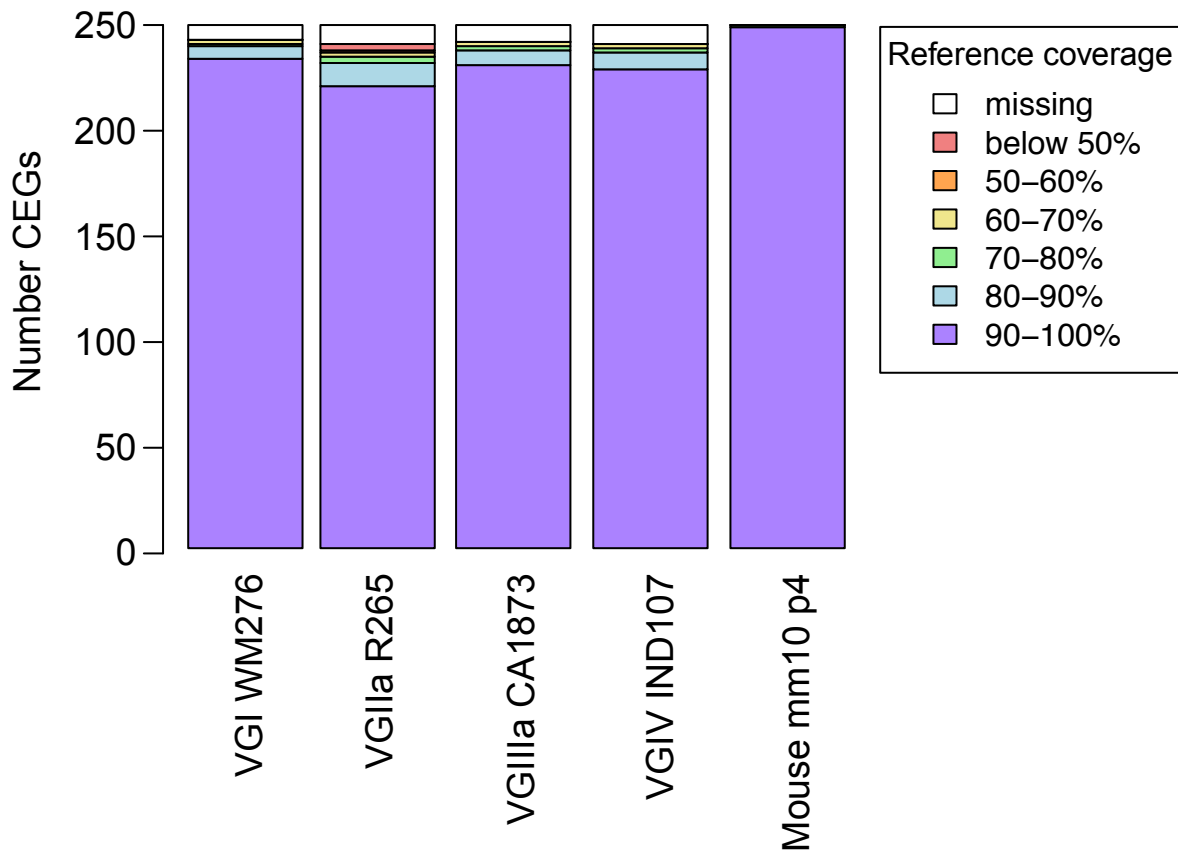

Supplement: FIG S1 [file sph006182675sf1.pdf]

# Mouse and *C. gattii* RNA (% of sequenced reads)

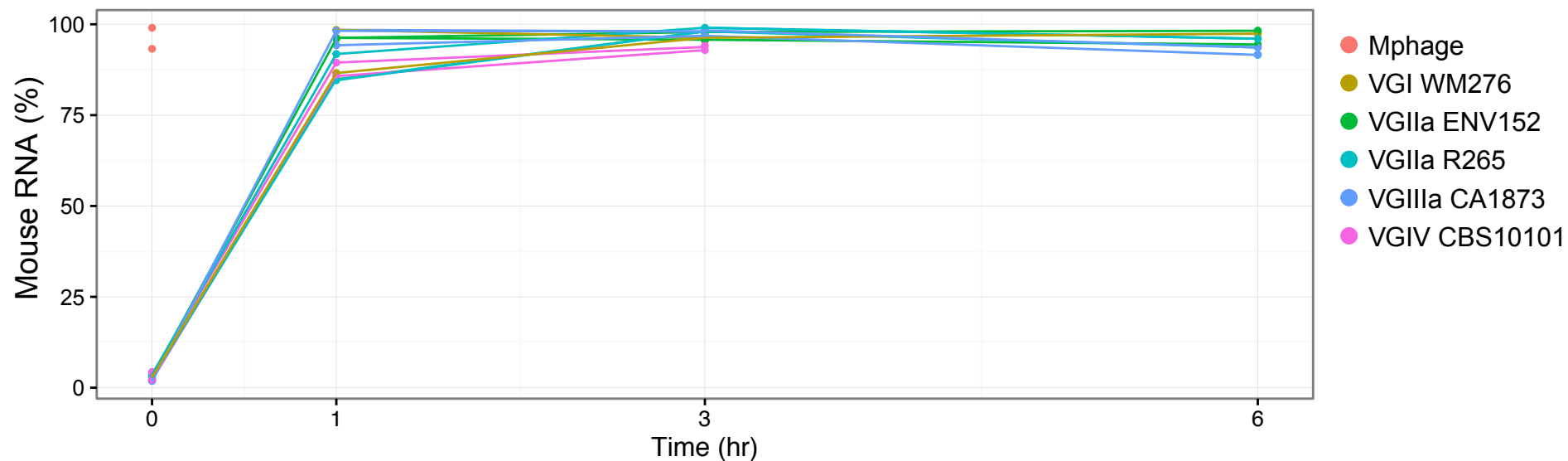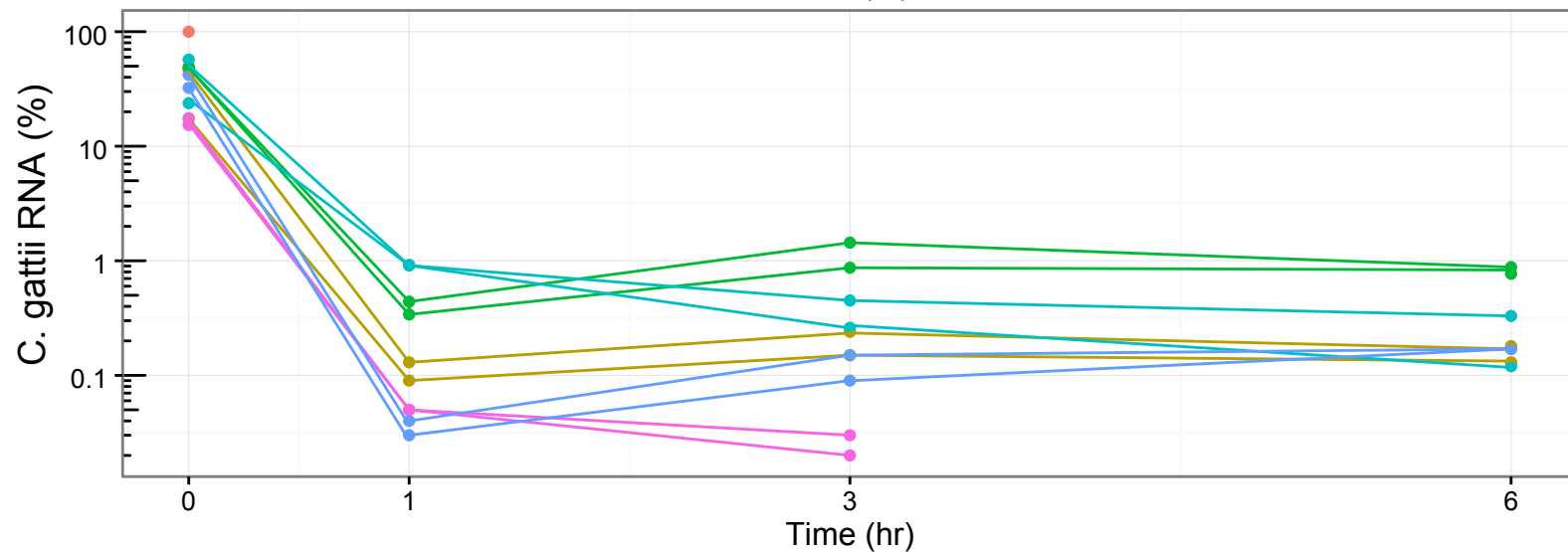

Supplement: FIG S2 [file sph006182675sf2.pdf]

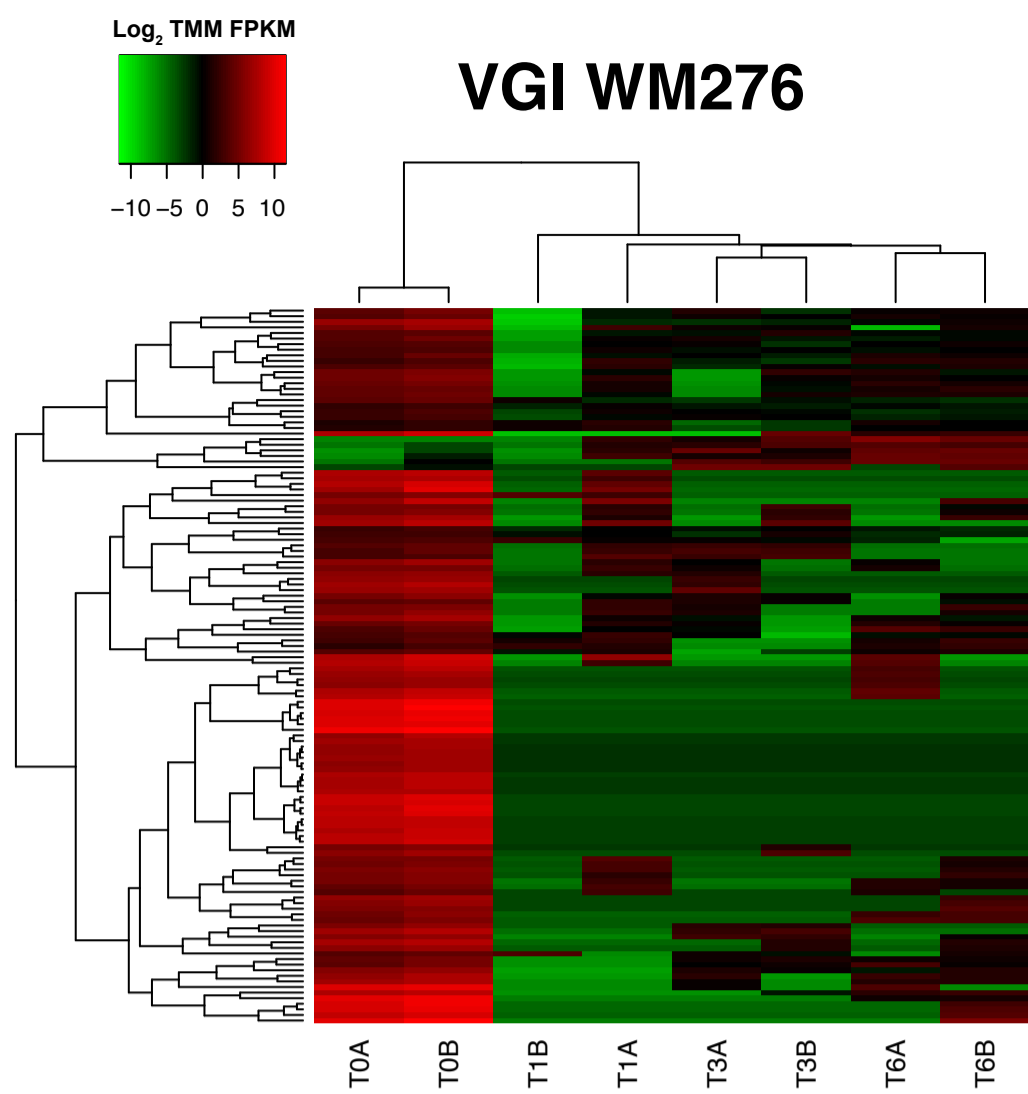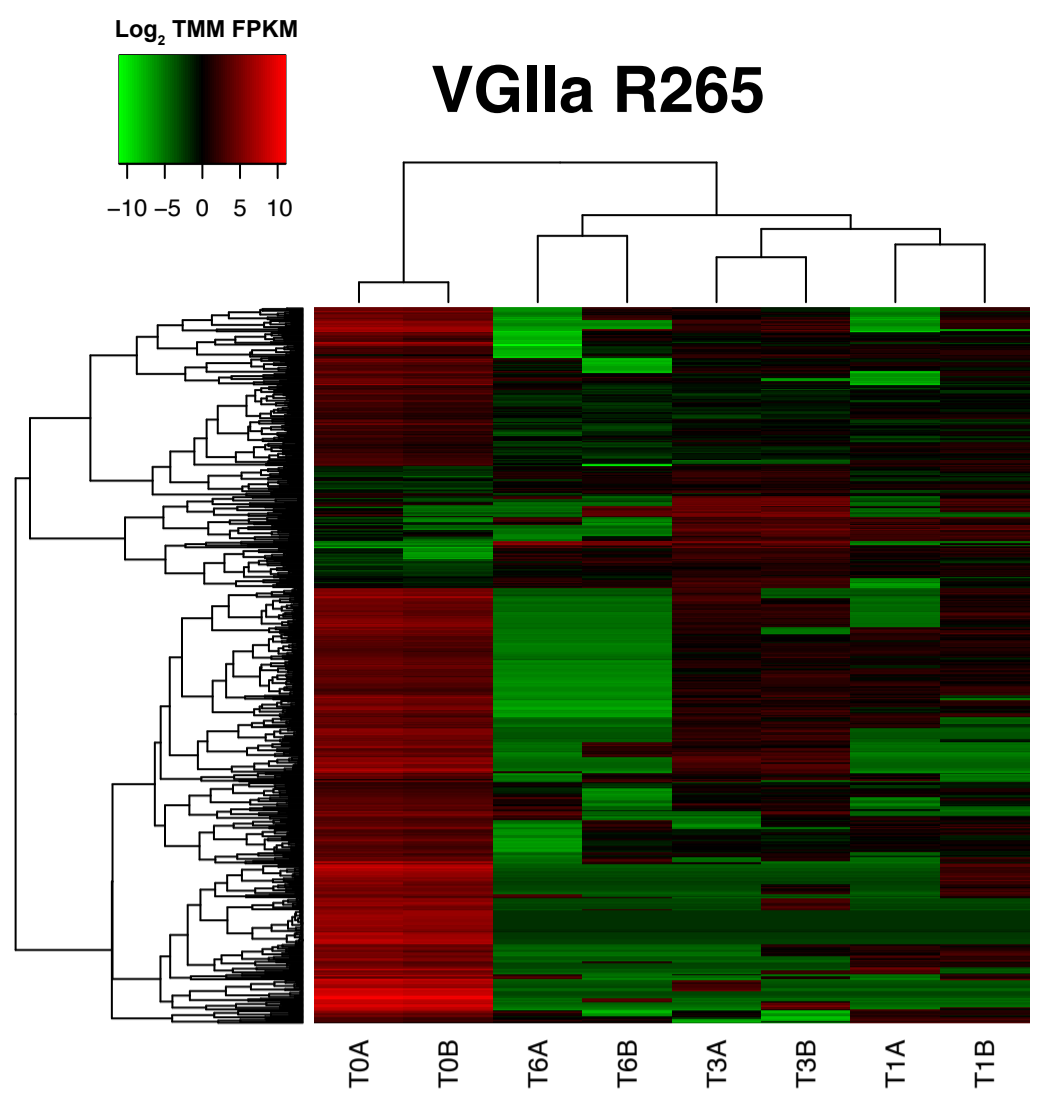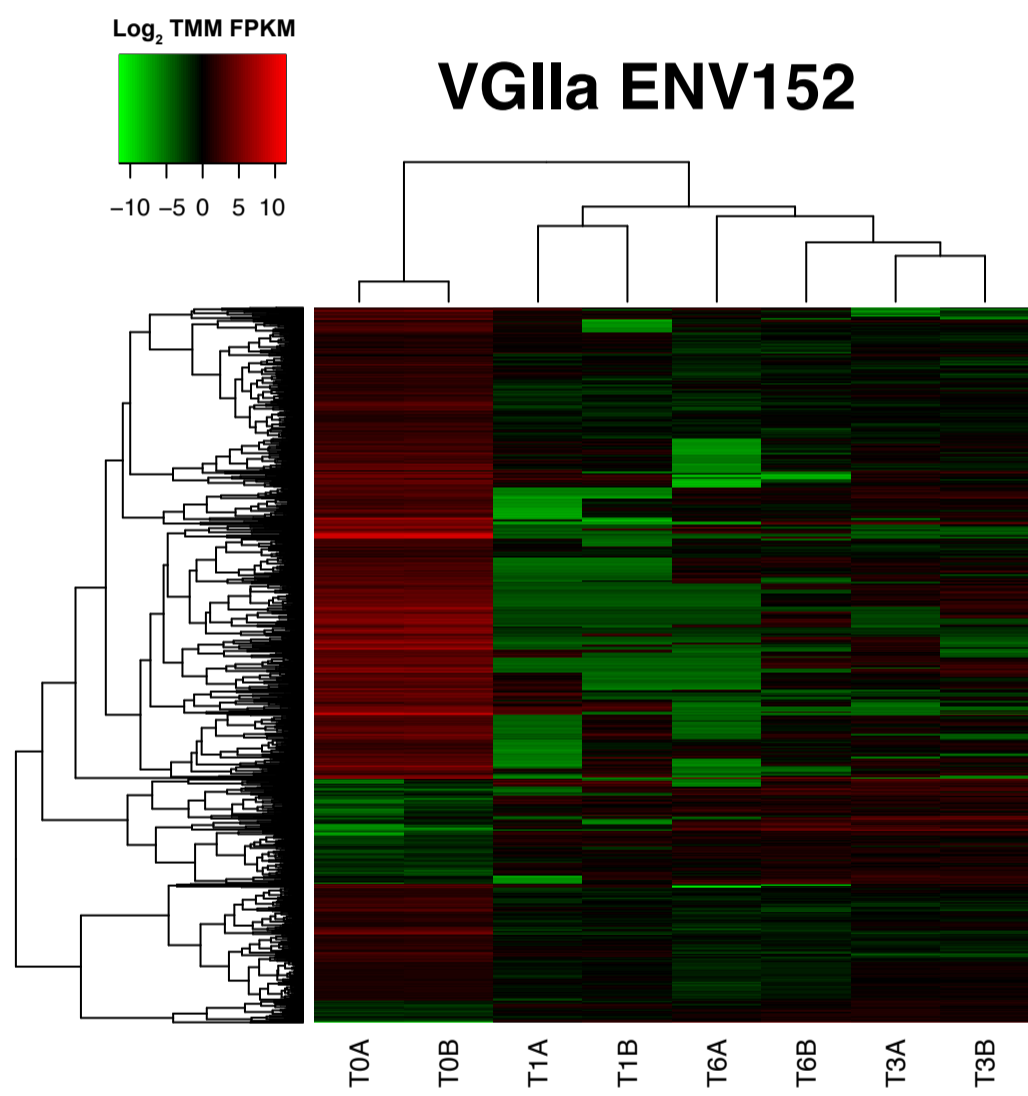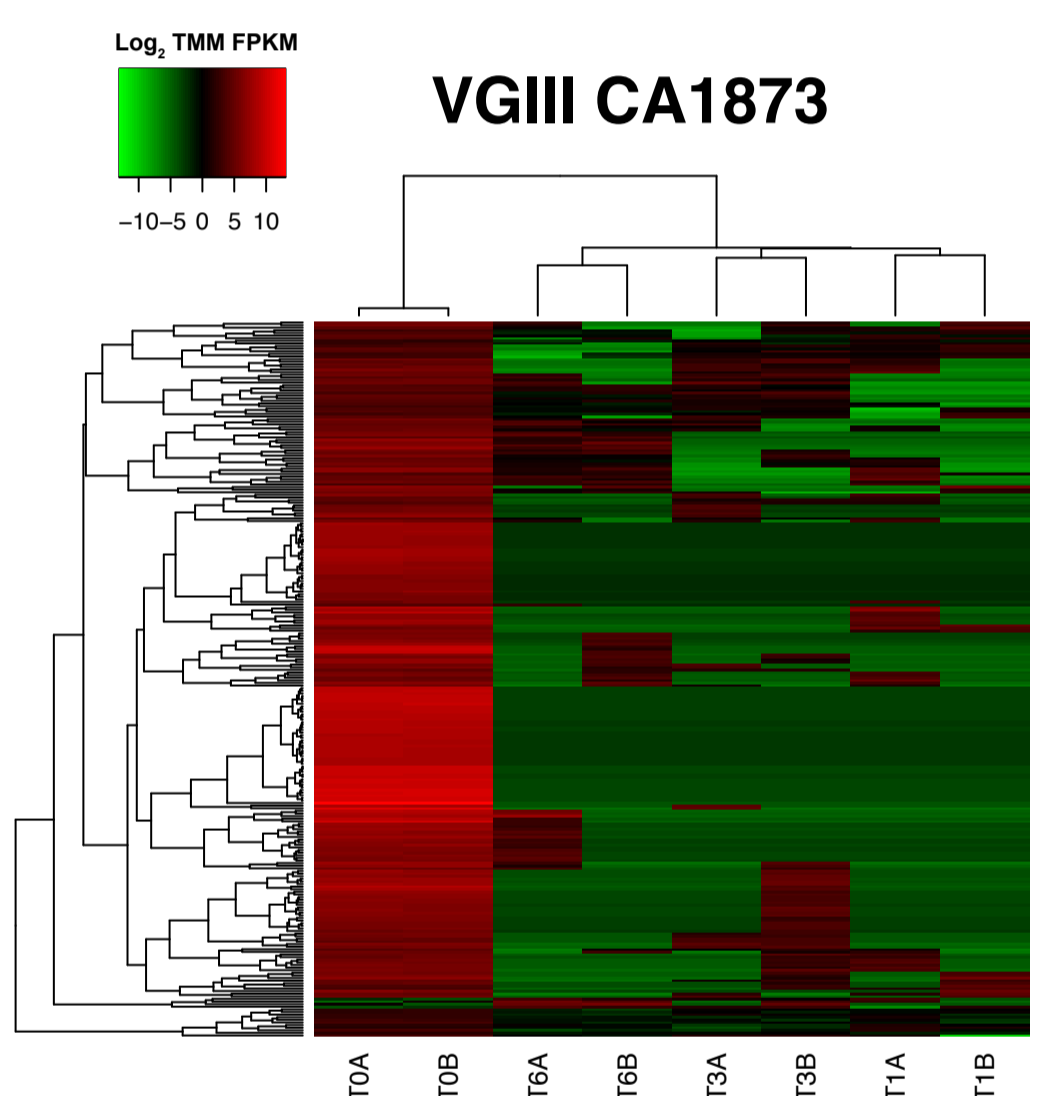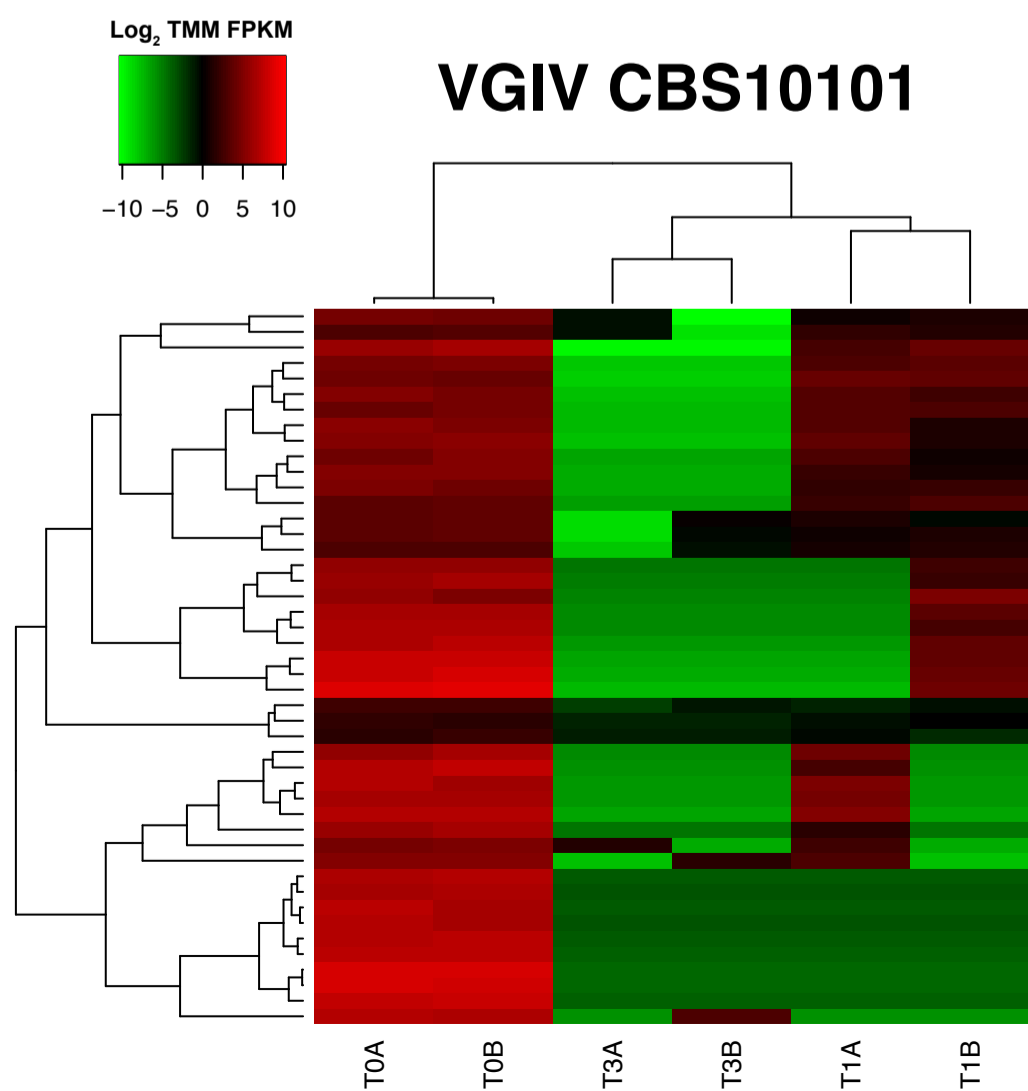

Supplement: FIG S3 [file sph006182675sf3.pdf]

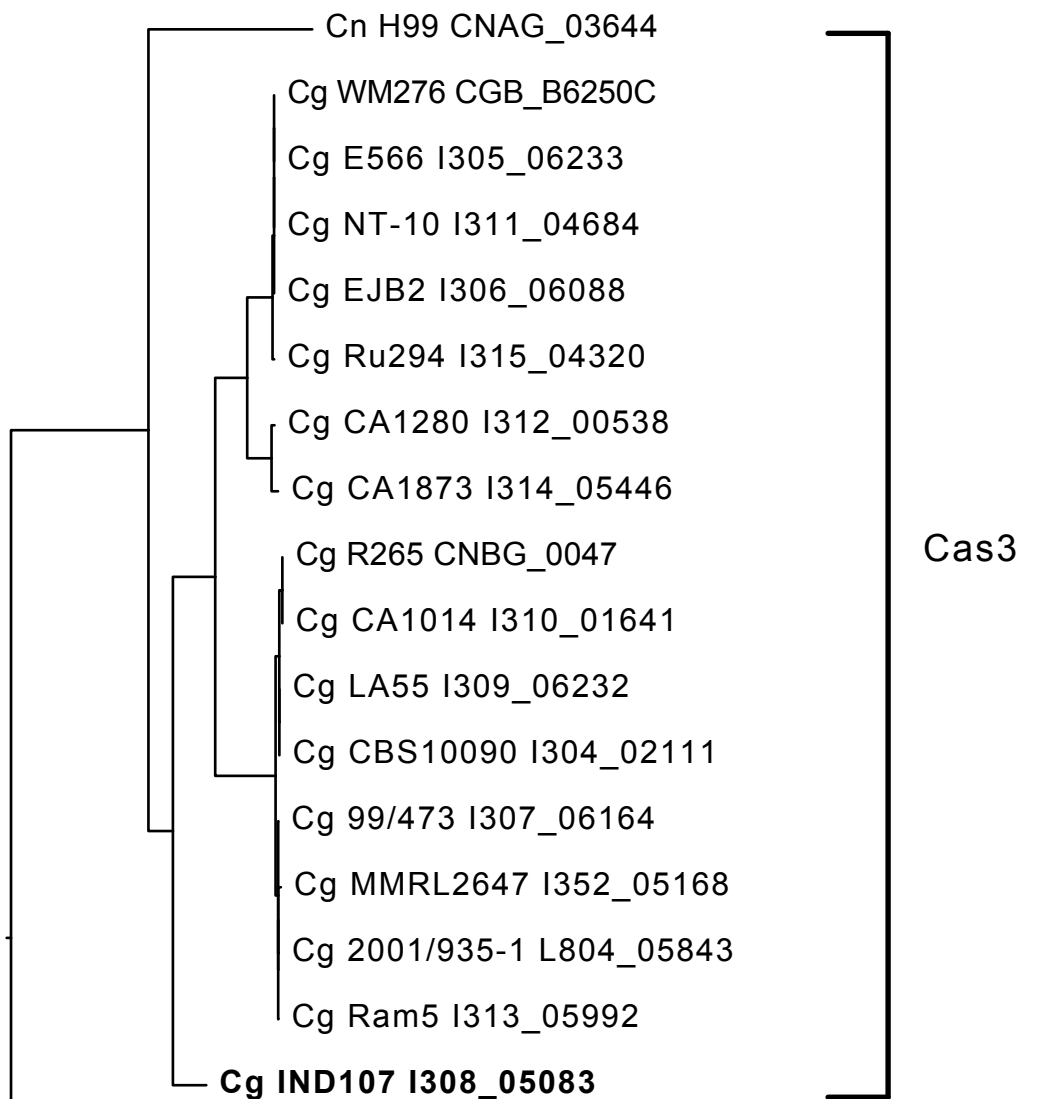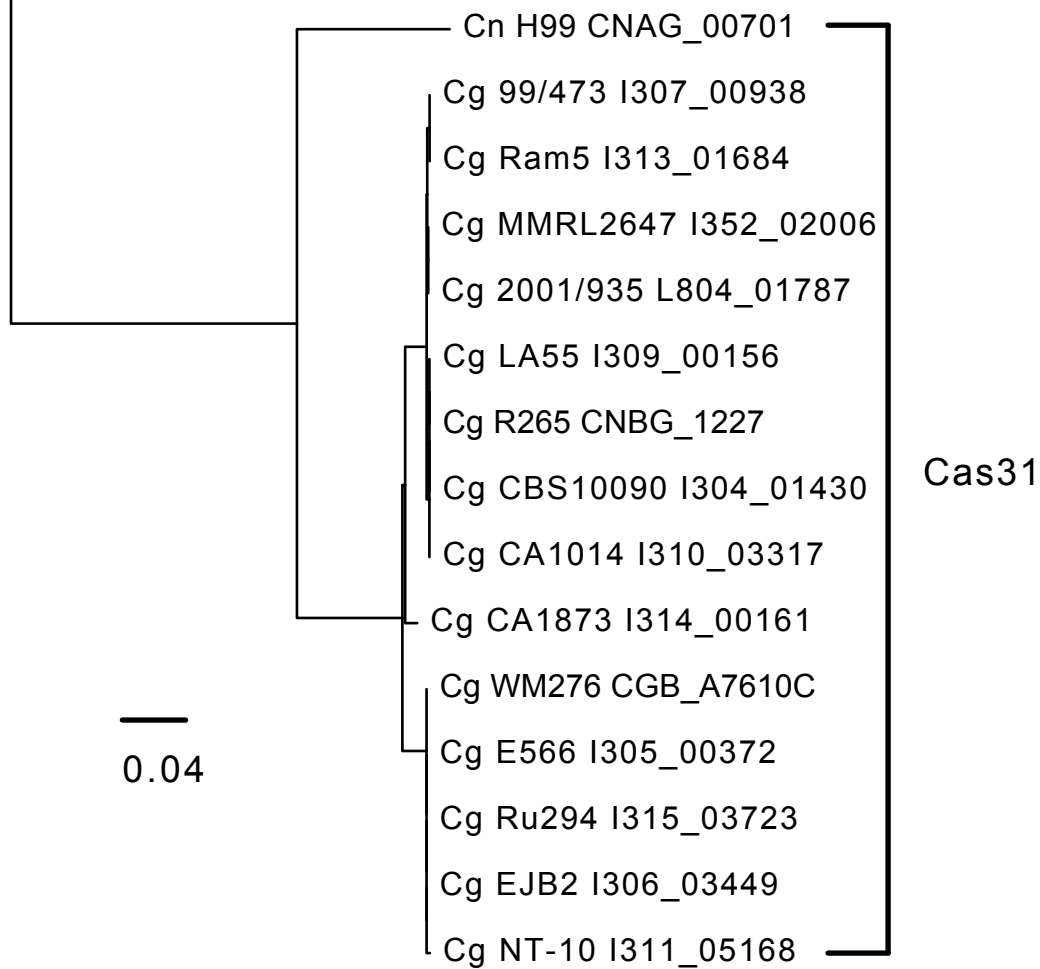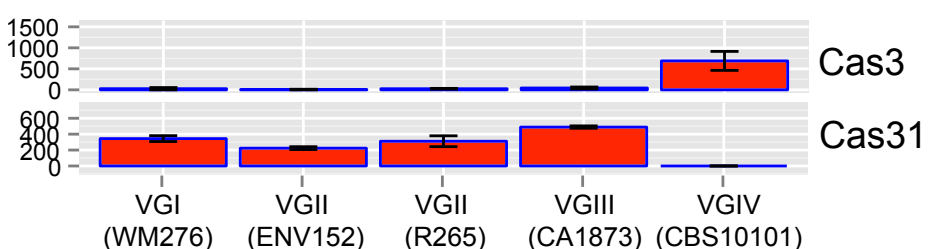

Supplement: FIG S5 [file sph006182675sf5.pdf]
