# Supplementary material for: Transcriptional Heterogeneity of Cryptococcus gattii VGII Compared with Non-VGII Lineages Underpins Key Pathogenicity Pathways
Source: mSphere. 2018 Oct 24;3(5):e00445-18. doi: 10.1128/mSphere.00445-18 (PMC6200987; doi:10.1128/mSphere.00445-18)
Supplement: FIG S4 [file sph006182675sf4.pdf]

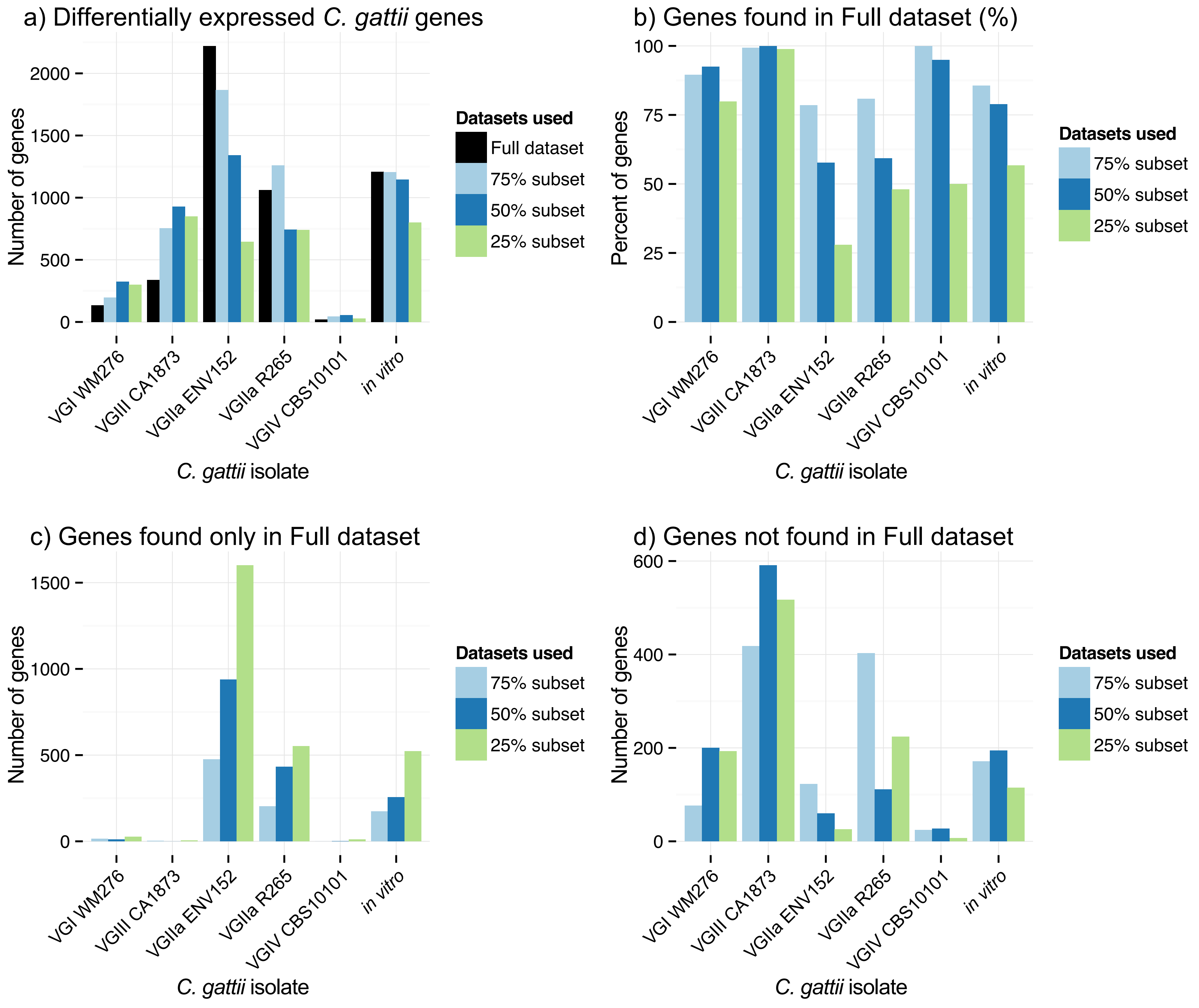

a) Differentially expressed C. gattii genes
Number of genes
0
500
1000
1500
2000
Datasets used
Full dataset
75% subset
50% subset
25% subset
VGI WM276
VGIII CA1873
VGIIa ENV152
VGIIa R265
VGIV CBS10101
in vitro
C. gattii isolate
b) Genes found in Full dataset (%)
Percent of genes
0
25
50
75
100
Datasets used
75% subset
50% subset
25% subset
VGI WM276
VGIII CA1873
VGIIa ENV152
VGIIa R265
VGIV CBS10101
in vitro
C. gattii isolate
c) Genes found only in Full dataset
Number of genes
0
500
1000
1500
Datasets used
75% subset
50% subset
25% subset
VGI WM276
VGIII CA1873
VGIIa ENV152
VGIIa R265
VGIV CBS10101
in vitro
C. gattii isolate
d) Genes not found in Full dataset
Number of genes
0
200
400
600
Datasets used
75% subset
50% subset
25% subset
VGI WM276
VGIII CA1873
VGIIa ENV152
VGIIa R265
VGIV CBS10101
in vitro
C. gattii isolate
